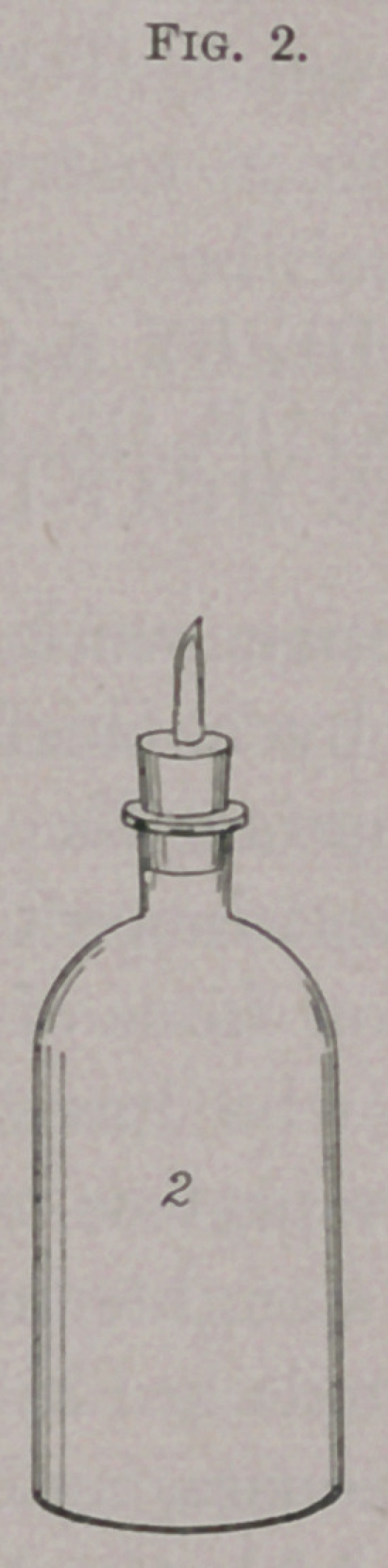# Tetanus Toxin and Antitoxin1Read at the thirty-sixth annual meeting of the American Veterinary Medical Association, September, 1899.

**Published:** 1899-10

**Authors:** Joseph McFarland, E. M. Ranck

**Affiliations:** Philadelphia, Pa.; Philadelphia, Pa.


### TETANUS TOXIN AND ANTITOXIN.[1]

BY JOSEPH MCFARLAND, M.D., AND E. M. RANCK, V.M.D.,
PHILADELPHIA, PA.

*Tetanus Produced Experimentally and Shown to be a Specific Disease.* In the first edition of Sternberg's *Manual of Bacteriology* the author says that in 1880 he "produced experimental tetanus in animals by injecting into them gutter-water and earth."

In 1884 it was shown by Carle and Rattone[2] that by the introduction of pus from human tetanus into animals they could be infected with the disease.

Later than this are the experiments of Nicolaier,[3] who introduced earth into small subcutaneous pockets at the root of the tail of white mice and house mice, guinea-pigs, and rabbits, and thus produced tetanus. Nicolaier, however, investigated the pus formed in these tetanic wounds, and, in addition to a large number of cocci which were present, invariably found a long, delicate bacillus, sometimes present in great numbers. This bacillus only occurred in the immediate neighborhood of the wound, and could not be followed into the tissues. Nicolaire also found that when the earth was heated to 190° C. for an hour, its ability to produce tetanus upon subcutaneous injection was entirely destroyed.

Pus taken from the tetanic wound was found to produce tetanus in animals into which it was introduced in 64 out of 88 cases. Nicolaier found the bacillus in certain earths with great regularity, but with equal regularity was unable to find it in earths from other sources. He never found it except in earth and in the pus from infected wounds. Later he found that the bacillus grew in culture media, but in spite of all his precautions and his many experiments he was never able to secure the bacillus, of whose specificity he was satisfied, in pure culture. In 1885, in an inaugural dissertation (Göttingen, 1885), he showed the tetanus bacillus to be widespread in the upper layers of the soil.

A little later Rosenbach[4] demonstrated the presence of Nicolaier's bacillus in the pus of human tetanus.

*The Isolation of the Specific Bacillus of Tetanus by Kitasato.* In

[1] Read at the thirty-sixth annual meeting of the American Veterinary Medical Association, September, 1899.

[2] Gior della R. Acad. di Med. di Torino, March, 1894, No. 3.

[3] Deutsche med. Wochenschrift, December, 1884, No. 52, p. 842.

[4] Archiv f. klin. Chirurgie, 1886, Bd. xxxiv, p. 306.

April, 1889, Kitasato[1] announced to the Eighteenth Congress of the German Surgical Society that he had successfully isolated the bacillus of Nicolaier in pure culture. The method of isolation depended upon two important facts not previously known: First, that the spores of the tetanus bacillus were resistant to heat, and, second, that the bacillus could not grow in an atmosphere containing oxygen. His description of the method is, in brief, as follows:

"The pus containing the tetanus bacillus is inoculated upon the surface of obliquely congealed agar-agar or blood-serum and placed in an incubator at 36° to 38° C. for twenty-four hours. All the microörganisms present grow, and the tetanus bacilli, which were few in number before, are now found to be quite numerous. The cultures are now stood in a water-bath and kept for one-half hour to an hour at 80° C., after which only the spores are found to be vital. A mouse inoculated with the culture, after this exposure to the high temperature, died of tetanus, showing that the spores were vital.

"An oseful of the cultures was now mixed with melted gelatin and some of the mixture poured upon plates in the ordinary way, while some was placed in closed flat dishes through which hydrogen gas was passed until a pure atmosphere of hydrogen was secured. All the cultures were now kept at 18° to 20° C.

"The colonies in the anaërobic cultures first appeared in a week's time. When they were examined microscopically at the end of ten days the tetanus bacillus with its typical 'end-spores' was found in pure culture. Animals inoculated from the pure culture died in two to three days."

The tetanus bacillus of Nicolaier and Kitasato is a rather long, slender, actively motile bacillus, with rounded ends and a characteristically large, round spore, situated at the end, so that the spore-containing bacilli resemble "drum-sticks," or, rather, pins.

The bacillus grows well in the ordinary culture media, *provided means are taken to entirely remove and withhold oxygen from the cultures.* Its growth is most rapid at 36° to 38° C., and is accelerated by the addition of 2 per cent. of grape-sugar. The cultures give off a horribly offensive odor, somewhat resembling onions. In the cultures, especially when grape-sugar is present, there is a copious generation of gas.

The bacillus is not very resistant to heat, but its spores can resist 80° C. maintained for an hour. They are, however, killed in five

[1] Deutsche med. Wochenschrift, 1889, No. 31.

minutes by a temperature of 100° C. They are not susceptible to germicides, and resist 5 per cent. carbolic acid for ten hours; in fifteen hours they are killed. Kitasato found that 5 per cent. carbolic acid with the addition of 0.5 per cent. of hydrochloric acid was fatal to spores in five hours.

The colonies formed by the bacillus in its growth are quite characteristic. Upon the surface of gelatin plates they somewhat resemble those of the bacillus subtilis, but are more pronouncedly filamentous. In gelatin punctures the growth takes place low down in the tube and consists of a central stalk from which innumerable fine branches are given off at right angles. The effect of this branching may be compared to a miniature fir-tree. The gelatin is slowly liquefied, the bacilli accumulating as a turbid precipitate at the bottom of the tube. A similar growth without the liquefaction occurs in thin 1 per cent. agar-agar. In both gelatin and agar-agar the gas production becomes very evident from bubbles that form in the media.

*Pathogenesis and Modes of Entrance.* From the experiments of Nicolaier, already quoted, it seems that the tetanus bacillus is a wide-spread saprophyte, living in the upper layers of the soil. It was formerly supposed that the bacilli which were found most plentifully in garden soil—*i. e.*, manured soil—were derived from the dung of the horse. This seems, however, not to be the case, for Le Dente has found that in the New Hebrides, where there are no horses, the soil is rich in tetanus bacilli.

How the tetanus bacillus, which is an obligatory anaërobic bacterium—that is, one that cannot live where air is present—can live in the upper layers of the soil exposed to the air, is an interesting and puzzling question. The only solution of the problem is to suppose that it grows in the soil as in the pus of wounds—in combination with some bacterium that rapidly absorbs oxygen.

Most warm-blooded animals are susceptible to the disease, the horse, the ass, the white mouse, and the guinea-pig especially so. Dogs are not killed by considerable-sized doses of the poison or of cultures of the bacilli. The majority of birds are immune. Frogs are said to become susceptible when their body-temperature is elevated.

The bacillus probably enters the body in nearly all cases through wounds that are made by objects previously soiled with earth, or pus from infected wounds, or wounds subsequently exposed to infection from these sources. The word earth, however, must not be interpreted to mean either fresh earth or soil only; it can be applied with equal readiness to various dust, as that in hay-lofts, which consists largely of pulverized soil, and for this reason must include also a large part of the dust of stables as well. The simple entrance of the tetanus bacillus may not be enough in itself to produce tetanus. This has partly been shown by Vaillard and Rouget, who found that when the tetanus bacilli were introduced minus their toxin they were promptly taken up by the phagocytic cells of the body and destroyed. If, on the other hand, the toxin was simultaneously introduced, or the tissues damaged by lactic acid or other chemical substances, the body-cells were injured and the bacilli were able to grow. The ability to grow in the body seems to depend upon one or the other of two conditions: Either the bacilli must enter a portion of tissues in which there is little or no oxygen —*i. e.*, where the blood-supply is feeble or poor—or its entrance must be accompanied by some other microörganism whose marked affinity for oxygen shall absorb all contained in the tissues so rapidly as to allow the tetanus bacillus to grow. The absence of these requisites probably explains why so many wounds inflicted with earth- and dust-soiled instruments are not followed by tetanus.

The inhibiting effect of oxygen upon the growth of the tetanus bacillus prevents the distribution of the organism throughout the body of the infected animal. The microbe is only found at the point of original inoculation. At this point, however, but little change (sometimes none) can be observed. There is, of course, the wound and in most instances a small area of suppuration, possibly caused by the tetanus bacillus itself, but more probably by other simultaneously introduced bacteria.

Seeing that the local lesion caused by the tetanus is of insignificance, and that the micro-organism does not disseminate throughout its host, we are led to the conclusion that the disease-producing capacity of the bacterium resides in the so-called poison that it elaborates. This is now proven to be correct.

*The Tetanus Toxin.* The poisonous substance responsible for the symptoms of tetanus was first sought by Brieger,[1] who, in 1887, succeeded in isolating from impure cultures of the bacillus (at that time no one had succeeded in securing it in pure culture) two substances—*tetanin* and *tetanotoxin*. The tetanin was found by Brieger, Kitasato, and Weyl to be toxic for small animals in very minute doses, producing in them symptoms of tetanus. The tetanus toxin, although it killed in small doses, did not produce the typical tetanic convulsions.

[1] Deutsche med. Wochenschrift, 1887, p. 303.

Later researches on the part of Brieger, Fraenkel,[1] and Kitasato,[2] have shown the real toxic substance of the tetanus bacillus to be a toxalbumin. This substance is so deadly that Kitasato found 0.00001 c.c. sufficient to kill a mouse, and of the dried precipitate of the toxalbumin obtained by Brieger and Cohn, 0.0000066 gramme per 100 grammes body-weight was sufficient to kill susceptible animals. In spite of the remarkable toxicity of this substance it was subsequently found that when further purified by dialysis the substance which could be secured in dry transparent scales, odorless and tasting like gum acacia, was readily soluble in water and so extremely poisonous that 0.00000005 gramme would cause the death of a mouse weighing 15 grammes, and one-fifth of the amount would suffice to produce violent tetanic symptoms from which, however, the animal would recover.

The toxicity of tetanus cultures, therefore, depends upon the presence of a toxalbumin elaborated by the bacilli. The figures above given certainly explain sufficiently why and how a few bacilli growing locally in a wound can produce in animals such marked general symptoms and a final fatal outcome.

The physiological action of the poison is interesting. Nicolaire was among the first to point out that the tonic convulsions of tetanus brought about by the introduction of earth beneath the skin began on that side and in that member near which inoculation was made. This would seem to suggest that the poison acted either upon the motor nerves or upon the muscles themselves. Experiments directed to determine this fact made by Gumprecht[3] have shown that this supposition is incorrect, for when the motor nerve is cut or curara administered, no convulsions occur.

From this it must be concluded that the poison acts through the central nervous system. Why the symptoms are first observed in the parts nearest to the point of inoculation is not clear. It may be that the irritation first reaches the central nervous system through the nerves themselves, and subsequently acts generally by distribution through the circulation.

The symptoms are not due simply to irritation of the central nervous system, but depend upon actual structural alteration of the nervous substances, as was pointed out by Goldschneider.[4]

It is important to note the rapidity with which these structural

[1] Berl. klin. Wochenschrift, 1890, No. 11.
[2] Zeitschrift f. Hyg., vol. x, p. 267.
[3] Habilitatiousschrift, Bohn, 1895.
[4] Zeitschrift f. klin. Med., Bd. xxxvi, Hyt. 1 and 2, p. 175.

changes are brought about, as this is a matter of vast importance in connection with the *antitoxic therapeusis* of the disease.

Kitasato found that if mice were inoculated with tetanus at the root of the tail, and, after a short time, the skin and surrounding tissues were excised or burned out, the eradication of the infected area would not save the animal's life *unless the operation was performed within an hour after the inoculation had been made.* Remembering that the development of the bacilli is a local one, and that they cannot live in tissues provided with free circulation, or in the blood, we are obliged to conclude that the death of the animals depended solely upon the toxin they elaborated. Similar to these results of Kitasato are those of Nocard upon sheep.

The rapidly fatal action of the toxin has led some to suppose that it is an enzyme or ferment—*i. e.*, is capable of increasing in the body of an animal into which it is injected. This is a matter exceedingly difficult of demonstration and has not yet been proven. Toward the fatal termination of the disease the amount of toxin in the blood is sufficient to be demonstrable by introducing some of it into other animals.

The toxic substance is produced in all cultures, but being perfectly soluble, is most easily secured from bouillon-cultures, though probably most active when secured from agar-agar cultures. The toxin is destroyed by heat and is decomposed by light.

To prepare the toxin in large quantities for experimental purposes, we have found the method suggested by one of us[6] [J. McF.] very convenient. It is, in brief, as follows :

An ordinary bottle is fitted with a perforated rubber cork containing a glass tube about three inches long. To the upper end of this, by means of a short rubber tube, a broken pipette of about 10 c.c. capacity, or a specially made tube with a bulb blown in it, is fastened. A cotton plug is placed in the upper end of the pipette or glass tube and it is sterilized by dry heat. The bottle is sterilized by dry heat, the rubber tubes by 5 per cent. carbolic-acid solution. The bottle is filled with bouillon all the way to the neck, and the apparatus rigged up as shown in the cut, and the whole thing sterilized by steam by the ordinarily employed intermittent method. During sterilization the expansion of the liquid within the bottle causes the contained fluid to flow up into the reservoir at *b* ; as it cools it retracts into the bottle again.

When ready for use the tubes are disconnected at *a*, and the

[1] Centralblatt f. Bakt. u. Parasitenk, April 25, 1896, xix. No. 14 and 15, p. 550.

bouillon inoculated through the tube in the rubber cork by means of a fine pipette (or the cork may be removed, though the danger of contamination is greater). After inoculation the tube *c* is held in a flame and drawn out to a long fine capillary tube, which is temporarily left open. The bottle is now stood up to its neck in a vessel of water kept for an hour at 80°, during which time the expansion of the bouillon causes some cubic centimetres of it to flow out of the capillary tube, and the heat drives out any air there may be in the bouillon. At the end of the hour the capillary glass tube is sealed in a flame and the culture is hermetically sealed.

FIG. 1. FIG. 2.

In an apparatus of this kind the tetanus bacillus grows well and produces a toxic substance of considerable strength. In our experience the toxicity of the culture is greatest in the third week.

The culture is killed by adding 2 per cent. of carbolic acid, allowing it to stand for twenty-four to forty-eight hours, then filtering through porcelain. The toxin so prepared is generally strong enough for 0.001 c.c. to kill an adult guinea-pig.

*Immunization Experiments.* The observation of numerous cases in human and veterinary medicine in which tetanus recovered led to the belief that animals could be immunized to the disease. The first experiments in this field were probably those of Kitasato,[1] who failed in his attempts to produce immunity though out of fifteen rabbits upon which he experimented he was able to produce a

[1] Zeitschrift f. Hygiene, Bd. x, p. 267.

high degree of immunity in six by the use of filtered cultures and trichloride of iodine. Tizzoni and Cattani[1] failed to produce immunity with live cultures, but succeeded in producing it in dogs by the use of filtered cultures the toxicity of which had been lessened by heat, mineral acid, etc.

Behring[2] found it possible to produce immunity to the toxin in rabbits and larger animals. In his experiments Behring found it necessary to destroy the extreme action of the toxin by adding to it 3 c.c. of a 1 per cent. solution of trichloride of iodine, as suggested by Kitasato. The immunity thus obtained lasted about two months.

(To be continued.)

## Figures and Tables

**Fig. 1. f1:**
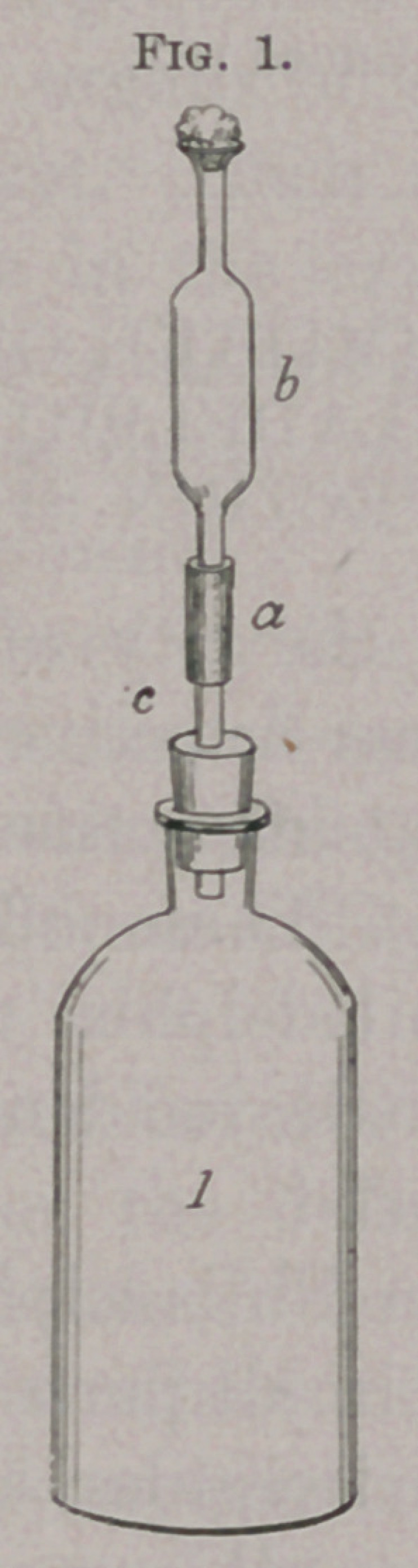


**Fig. 2. f2:**